# Probing molecular dynamics with hyperpolarized ultrafast Laplace NMR using a low-field, single-sided magnet†
†Electronic supplementary information (ESI) available: Experimental details and numerical results pertaining to the *D*–*T*_2_ maps. See DOI: 10.1039/c8sc01329b


**DOI:** 10.1039/c8sc01329b

**Published:** 2018-06-28

**Authors:** Jared N. King, Alfredo Fallorina, Justin Yu, Guannan Zhang, Ville-Veikko Telkki, Christian Hilty, Tyler Meldrum

**Affiliations:** a Department of Chemistry , The College of William & Mary , Williamsburg , Virginia 23187-8795 , USA . Email: tkmeldrum@wm.edu; b Department of Chemistry , Texas A&M University , 3255 TAMU , College Station , Texas 77843 , USA; c NMR Research Unit , Faculty of Science , University of Oulu , 90014 Oulu , Finland

## Abstract

Laplace NMR (LNMR) offers deep insights on diffusional and rotational motion of molecules. The so-called “ultrafast” approach, based on spatial data encoding, enables one to carry out a multidimensional LNMR experiment in a single scan, providing from 10 to 1000-fold acceleration of the experiment. Here, we demonstrate the feasibility of ultrafast diffusion–*T*_2_ relaxation correlation (*D*–*T*_2_) measurements with a mobile, low-field, relatively low-cost, single-sided NMR magnet. We show that the method can probe a broad range of diffusion coefficients (at least from 10^–8^ to 10^–12^ m^2^ s^–1^) and reveal multiple components of fluids in heterogeneous materials. The single-scan approach is demonstrably compatible with nuclear spin hyperpolarization techniques because the time-consuming hyperpolarization process does not need to be repeated. Using dynamic nuclear polarization (DNP), we improved the NMR sensitivity of water molecules by a factor of 10^5^ relative to non-hyperpolarized NMR in the 0.3 T field of the single-sided magnet. This enabled us to acquire a *D*–*T*_2_ map in a single, 22 ms scan, despite the low field and relatively low mole fraction (0.003) of hyperpolarized water. Consequently, low-field, hyperpolarized ultrafast LNMR offers significant prospects for advanced, mobile, low-cost and high-sensitivity chemical and medical analysis.

## Abstract 2

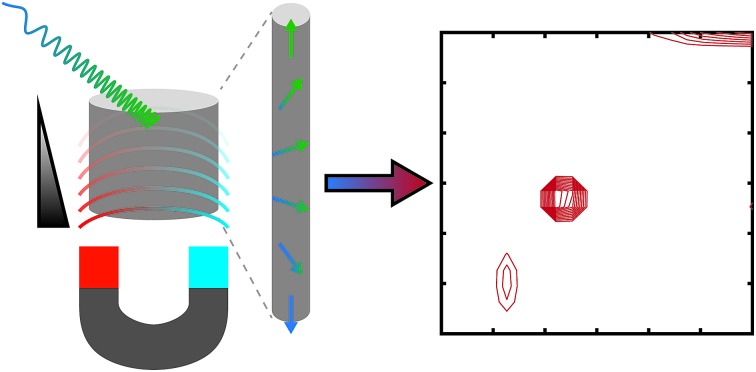
Ultrafast NMR measurements of diffusion and *T*_2_ relaxation reveal physical properties of samples and are compatible with hyperpolarization-based signal enhancement.

## Introduction

Multidimensional NMR experiments, such as those used to determine the 3D structures of proteins,^1^ are indispensable in modern chemical analysis because they significantly improve the resolution relative to 1D experiments and can correlate different NMR parameters. Traditional multidimensional experiments require a large number of repetitions in order to collect the indirect dimensions, resulting in long experiment times, ranging from minutes to even days.^2^ Ultrafast NMR spectroscopy, introduced in 2002,^3^–^6^ can deliver 2D NMR spectra in a single scan. The method is based on the encoding of various evolution periods into spatially distinct regions of the sample using simultaneous gradient fields and adiabatic frequency-swept pulses. The data is read by applying the principles of magnetic resonance imaging (MRI). The method enables the investigation of fast molecular processes in real time. It also facilitates the use of modern nuclear spin hyperpolarization techniques, such as dynamic nuclear polarization (DNP);^7^ parahydrogen-based methods,^8^ including signal amplification by reversible exchange (SABRE);^9^ and spin-exchange optical pumping (SEOP) of noble gases.^10^ Though each of these hyperpolarization methods alone can boost the sensitivity of the experiment by several orders of magnitude, they are more useful in combination with ultrafast methods that do not require multiple indirect points as the (time-consuming) process of generating hyperpolarization does not need to be repeated.^11^–^13^ So far, ultrafast NMR experiments have mainly been carried out on high-field NMR instruments, but some recent applications with a high-resolution benchtop instrument equipped with a gradient coil have also been reported.^14^–^16^

Single-sided NMR has been used in the past two decades to characterize physical properties of various samples, including paintings,^17^,^18^ coatings,^19^,^20^ buildings and building materials,^21^,^22^ polymers and elastomers,^23^,^24^ food,^25^,^26^ and even humans.^27^–^29^ Single-sided magnets have several advantages over traditional NMR hardware: they are portable; they are less costly; and they impose only loose restrictions on sample geometry, allowing non-invasive measurements of large objects like paintings and buildings. However, unlike in traditional high-field NMR or high-resolution benchtop instruments, chemical shift information is inaccessible in the strongly inhomogeneous magnetic field of single-sided devices. Instead, single-sided devices characterize physical properties of materials by measuring spin–lattice (*T*_1_) and spin–spin (*T*_2_) relaxation, molecular self-diffusion coefficients (*D*), and other NMR parameters that rely on NMR signal attenuation.^30^ As with high-field NMR, multidimensional data (*e.g.*, *T*_1_–*T*_2_, *D*–*T*_2_)^31^–^33^ can be determined by repeating measurements with different evolution time delays and employing an appropriate data transformation. Whereas NMR spectroscopy relies on the Fourier transform to connect the time and frequency domains, NMR relaxometry and diffusometry use the inverse Laplace transform (ILT) to extract relaxation time and diffusion coefficient distributions from the measurement data;^31^ both relaxation and diffusion measurements are a subset of Laplace NMR (LNMR). Only since the early 2000s have reliable ILT algorithms been available to researchers, enabling robust interpretation of NMR relaxation measurements.^34^–^36^ Previous research has demonstrated single-shot determination of diffusion coefficients by encoding relaxation into the shape of the NMR signal in the time domain;^37^–^39^ however, these experiments are typically done in magnetic field gradients much weaker than those provided by single-sided magnets and they have not generated simultaneous correlation between *T*_2_ and *D*.

Recently, it has been shown that multidimensional LNMR data can be measured in a single scan using a high-field spectrometer.^40^–^42^ Similar to ultrafast NMR spectroscopy^3^ and to 1D diffusion^43^–^46^ and relaxation experiments,^47^,^48^ this so-called ultrafast LNMR method is based on spatial encoding of multidimensional data. We have previously shown that ultrafast LNMR is compatible with single-sided NMR, by exploiting the field gradient intrinsic to single-sided magnets for spatial encoding.^49^ We measured ultrafast *T*_1_–*T*_2_ maps that were in good agreement with the traditional method. However, extensive signal averaging was needed due to low thermal nuclear spin polarization and detection sensitivity in the low-field instrument.

In this article, we demonstrate that ultrafast diffusion–*T*_2_ relaxation correlation (*D*–*T*_2_) measurements are feasible with a single-sided magnet. Diffusion and relaxation measurements excel at measuring pore-size distributions and tortuosity within a sample, leading to applications in oil logging, building materials analysis, food science, *etc.*^21^,^25^,^50^,^51^ Single-sided magnets have a magnetic field gradient that is much stronger than in standard high-field NMR probes and comparable with state-of-the-art diffusion probes. Therefore, one can probe very small molecular self-diffusion coefficients, even down to 10^–14^ m^2^ s^–1^.^52^ Furthermore, we demonstrate the combination of DNP hyperpolarization with the ultrafast approach, making single-scan *D*–*T*_2_ experiments feasible; this advancement is ground-breaking in low-field NMR.

## Theory

Diffusion measurements require the encoding of the positions of nuclear spins using a field gradient. In traditional pulsed-field gradient measurements, the strength of the gradient field is varied in order to observe the signal attenuation due to diffusion.^31^ Various time delays within the pulse sequence are kept constant in order to ensure that relaxation does not affect the shape of the decay curve. However, the field gradient of single-sided NMR instruments cannot be varied. Therefore, the length of the “effective gradient pulse” (*i.e.*, the time of the spin system evolution under the magnetic field gradient—there is no pulsed gradient) must be varied in order to encode the appropriate signal decay. In this constant-gradient case, the effect of relaxation can be mitigated using relaxation-compensated pulse sequences.^53^–^56^

**Fig. 1:**
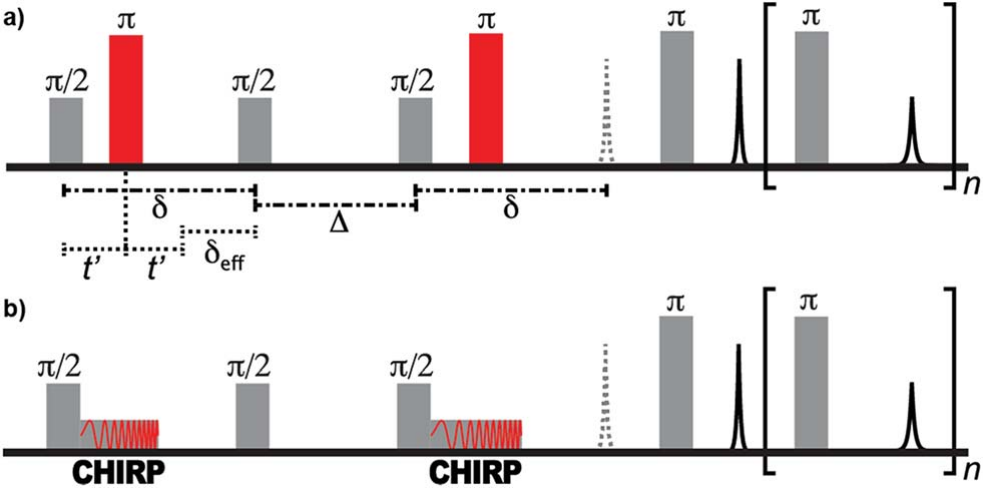
(a) Traditional relaxation-compensated *D*–*T*_2_ pulse sequence. (b) Ultrafast *D*–*T*_2_ pulse sequence. In the single-sided instrument, a constant field gradient is present throughout the experiment.

The pulse sequences of traditional and ultrafast *D*–*T*_2_ experiments used in this work are shown in Fig. 1. The traditional sequence includes a stimulated-echo based relaxation-compensated diffusion encoding.^54^ The delay *δ* between the π/2 pulses is kept constant, and the effective length of the gradient, *δ*_eff_, is varied by adjusting the onset of the π refocusing pulse between the π/2 pulses. When the effect of relaxation is neglected, the amplitude of the signal after the diffusion encoding depends exponentially on the molecular self-diffusion coefficient, *D*, as *S*(*δ*_eff_) = exp[–*b*(*δ*_eff_)*D*].^54^,^55^ The constant *b* depends on several experimental parameters as:1
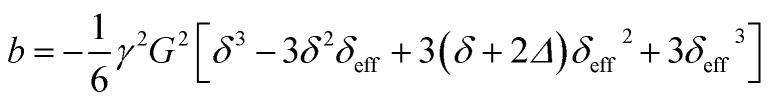
here, *γ* is the gyromagnetic ratio, *G* is the gradient strength, and the pulse sequence delays (*δ*, *δ*_eff_, *Δ*) are defined above. The diffusion encoding is followed by the Carr–Purcell–Meiboom–Gill (CPMG) acquisition that both encodes *T*_2_ relaxation and acquires the signal.^57^,^58^

The ultrafast sequence is otherwise identical to the traditional sequence, but the hard π refocusing pulses that encode diffusion are replaced with frequency-swept CHIRP pulses.^41^ The linear frequency sweep of these pulses results in spins at different positions within the magnetic field gradient of the single-sided instrument becoming inverted at different times. Spins that experience the π rotation at the end of the CHIRP pulse (*i.e.*, at a higher frequency) have *δ*_eff_ = 0. In contrast, spins that experience the rotation at the beginning of the pulse have *δ*_eff_ = *δ*. Between these extremes, the effective duration of the applied field gradient is linearly dependent on position. Consequently, at the end of the diffusion encoding, the magnitude of the transverse magnetization as a function of position along the field gradient is equivalent to the diffusion decay curve observed in the traditional (non-frequency-swept) experiment. This magnetization profile is read by applying the principles of MRI throughout the CPMG refocusing/acquisition loop, during which the gradient of the single-sided magnet acts as a read gradient. In the ultrafast experiment, the full *D*–*T*_2_ data is equivalent to the traditional experiment and is collected in a single scan.^41^

**Fig. 2:**
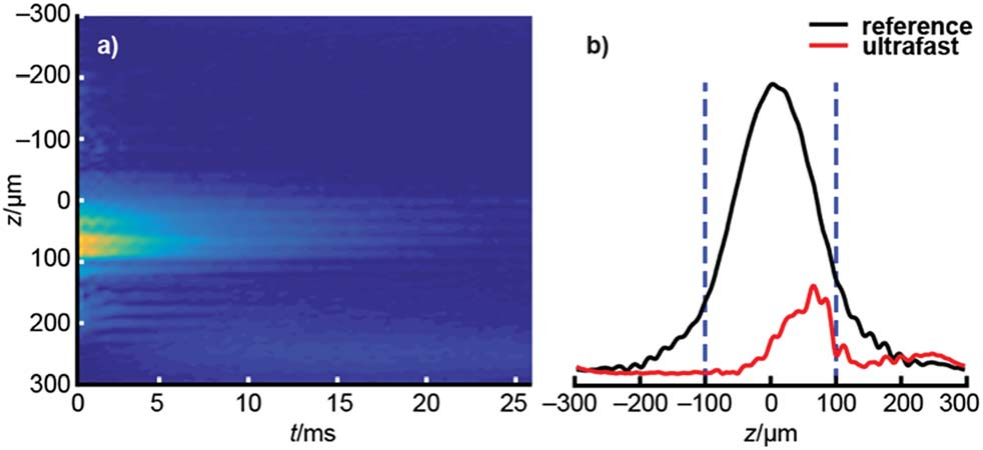
(a) The raw data of the ultrafast *D*–*T*_2_ experiment of hyperpolarized water. The vertical axis represents the spatial dimension, acquired following Fourier transform of each echo individually, while the horizontal axis represents the time at which each echo is acquired. The time projection (sum of all columns) is shown on the right. (b) Comparison of the signal profiles of the ultrafast (red) and reference (black) experiments. In the post processing, the ultrafast data was divided by the reference profile in order to eliminate the effect of inhomogeneous sensitivity of the rf coil across the sample. Dashed blue bars indicate the spatial bandwidth of the CHIRP pulse, 200 μm in this example.

After acquiring the ultrafast *D*–*T*_2_ data, each echo of the dataset is Fourier transformed, producing a profile that shows the effect of spatially encoding *δ*_eff_. All these echoes together form a raw 2D, revealing the signal intensity as a function of position and the time at which each echo was acquired (see Fig. 2a). Since the frequency sensitivity of the radiofrequency coil is non-uniform, making the spatial sensitivity also non-uniform, the raw dataset is divided by the coil sensitivity profile (see Fig. 2b), itself measured as a reference experiment in which *δ*_eff_ = 0 to eliminate the effect of the sensitivity inhomogeneity.^41^ The data outside the relevant CHIRP sweep region is removed, and the resulting dataset is subjected to an appropriate 2D inverse Laplace transform to produce the *D*–*T*_2_ map of interest.^36^

## Experimental

### Samples

Samples included glycerol, ethylene glycol, deionized water doped with copper(ii) sulfate (0.1% by weight), and 4 Å molecular sieves (particle diameter 3–4 mm) in a deionized water bath. These samples were chosen to have a broad range of diffusion coefficients. All materials were used as received (Sigma-Aldrich, St. Louis, MO).

### NMR experiments

The experiments were carried out using a PM25 NMR-MOUSE (0.3 T, 13 MHz ^1^H frequency) single-sided magnet with a specified gradient of 6.59 T m^–1^ (Magritek; Wellington, New Zealand) and a Scout spectrometer (Tecmag; Houston, TX).

Ultrafast *D*–*T*_2_ pulse sequences were designed with *δ* = 0.4–5 ms and *Δ* = 2–40 ms. The reference measurements (to evaluate the rf coil sensitivity) were implemented with a hard π pulse in the middle of the *δ* period, while the UF sequences had an adiabatic, frequency-swept CHIRP inversion pulse. The hard π pulse was calibrated at 6 μs (*γB*_1_/(2π) = 83.4 kHz). The power of the CHIRP pulses was varied depending on the duration of the CHIRP pulse; however, all had a maximum pulse power of *γB*_1_/(2π) = 48.1 kHz. The CHIRP pulse amplitude profile was shaped with a WURST function.^5^,^59^ The frequency sweep range of the CHIRP pulses was 84.2 kHz, corresponding to a spatial bandwidth of 300 μm. The number of echoes, echo time, and repetition time were varied as appropriate to capture the full decay and allow for a complete return to thermal equilibrium after each acquisition. The dwell time that was used for acquisition was 4 μs (2 μs for DI water in molecular sieves) corresponding to a spatial bandwidth of 250 kHz or 890 μm (500 kHz or 1780 μm); this is much larger than the corresponding spatial bandwidth of the rf coil (∼350 μm) to prevent aliasing artifacts. Other experimental parameters are given in Tables S1 (ultrafast experiments) and S2† (traditional experiments).

The sample for hyperpolarization consisted of 50 μL ethylene glycol in H_2_O (v/v 2 : 3) with 15 mM of 4-hydroxy-2,2,6,6-tetramethylpiperidine 1-oxyl radical (TEMPOL; Sigma-Aldrich, St. Louis, MO). Hyperpolarization was performed in a HyperSense DNP polarizer (Oxford Instruments; Abingdon, UK). For this purpose, the sample was irradiated with 100 mW of microwaves at a frequency of 94.005 GHz and a temperature of 1.4 K, in a field of 3.35 T for 30 min. Subsequently, the hyperpolarized sample was rapidly dissolved in 8 mL of water preheated until a pressure of 10 bar was reached. The dissolved sample was transferred into an injection loop in a sample injector described previously.^60^ Using water from a high-pressure syringe pump (model 1000D, Teledyne ISCO; Lincoln, NE), the sample was driven into a custom sample holder (Fig. S1†) that was designed to accommodate the maximum amount of liquid in the sensitive volume of the single-sided magnet. Flow was stopped by simultaneously switching two multi-port valves located at the inlet and the outlet of the sample holder, thereby trapping the injected liquid. The time elapsed from the beginning of the injection to the start of NMR measurement was 595 ms.

DNP experiments were performed with the same single-sided magnet using similar parameters as the non-hyperpolarized experiments, except that a lower-power rf amplifier, available on-site with the DNP apparatus, was used resulting in different pulse lengths/powers. Consequently, the hard π pulse was calibrated at 12 μs (*γB*_1_/(2π) = 41.7 kHz), the CHIRP pulse had a strength of *γB*_1_/(2π) = 16.7 kHz that was ramped linearly from/to 0 kHz at the beginning and end of the pulse, and the CHIRP pulse frequency sweep spanned 56.1 kHz (spatial bandwidth of 200 μm). In addition, the dwell time used for acquisition was 6 μs, corresponding to a bandwidth of 167 kHz (594 μm).

### Data processing

Data were processed using a custom Matlab script that calculates SNR, Fourier transforms each echo of the data, normalizes the CHIRP-encoded echoes against the reference echoes, then exports the appropriate data range. These results are subject to a 2D ILT using a Matlab program (provided by Petrik Galvosas, New Zealand). Peak positions within the *D*–*T*_2_ map are computed with another Matlab script that identifies *D* and *T*_2_ at each peak's maximum intensity (the reported values), as well as *D* and *T*_2_ at half of each peak's height, both above and below the maximum value. These values are used to approximate the error in *T*_2_ and *D*, though the values are upper limits because of the logarithmic axis in which ILTs are performed. All custom Matlab scripts are available *via* the corresponding author's institutional repository.

## Results

### Ultrafast measurements

**Fig. 3:**
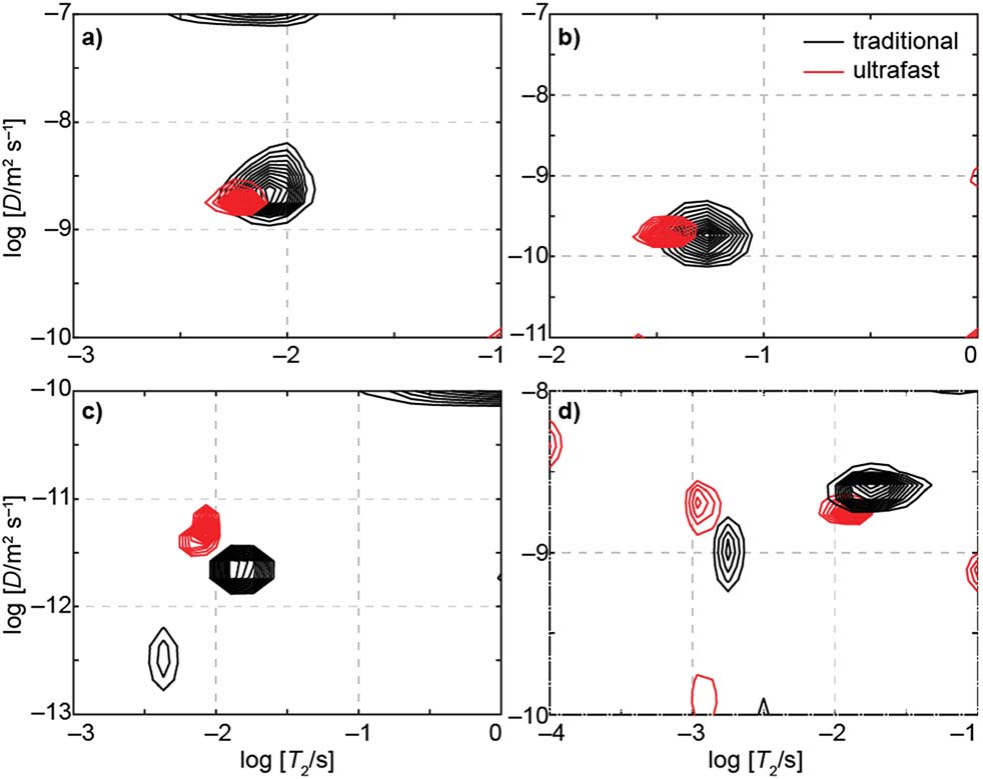
Traditional (black) and ultrafast (red) *D*–*T*_2_ correlation maps of (a) water doped with copper(ii) sulfate, (b) ethylene glycol, (c) glycerol, and (d) water in 4 Å molecular sieves. Values for *D* and *T*_2_ for these samples are given in Table S3.†

Fig. 3 displays *D*–*T*_2_ maps of four samples measured using both the traditional and ultrafast methods. Two samples, ethylene glycol and doped water, demonstrate quantitative agreement between ultrafast and traditional measurements. Both of these samples produce only one peak with diffusion coefficients (see values in Table S3†) that are in agreement with the values reported in the literature within the experimental error (water: *D* ≈ 2.2 × 10^–9^ m^2^ s^–1^; ethylene glycol: *D* ≈ 1 × 10^–10^ m^2^ s^–1^).^61^,^62^ Two other samples, glycerol and molecular sieves in a water bath, produce ultrafast results that are similar to but do not completely overlap with the traditional results. Glycerol has a very small molecular self-diffusion coefficient at room temperature (2.5 × 10^–12^ m^2^ s^–1^),^63^ three orders of magnitude smaller than that of water—this small *D* combined with short relaxation times (*T*_1_ ∼ 30 ms, *T*_2_ ∼ 10 ms) makes the measurement challenging (the encoding in this case may be improved by using a different single-sided magnet with a stronger field gradient). Nevertheless, both the ultrafast and traditional measurements are close to the literature value. The *D*–*T*_2_ maps of water in molecular sieves include two components: one with smaller *T*_2_ and *D* values arising from water inside the pores and the other from bulk-like water in between the macroscopic particles themselves. The small differences in the peak positions may originate from imperfections in spatial encoding due to the heterogeneity of the material but are nonetheless clearly resolved in ultrafast measurements.

In addition to the true signals, there are some low-amplitude artificial signals present both in the conventional and ultrafast maps; these are particularly clear close to the edges of the maps. These signals are due to the low signal-to-noise ratio in low-field data and to the ill-posed nature of the inverse Laplace transformation.^33^ In addition, the *T*_2_ values in the ultrafast maps appear slightly smaller than in the traditional maps. This may result from the fact that most of the encoded magnetization is located away from the center of the sensitive region of the NMR coil (see Fig. 2b). In those regions, the π pulses of the CPMG loop are imperfect, leading to additional signal decay. On the other hand, this effect seems to be quite small, as the changes are within the line widths of the peaks themselves (see Table S3† for numerical results).

Because of low thermal nuclear polarization, especially so at a low field strength, a large number of scans was accumulated for both the ultrafast and traditional experiments. Even so, the ultrafast approach resulted in enhanced efficiency. For example, the traditional *D*–*T*_2_ measurement of doped water was done with 256 scans for each of the 25 indirect points, resulting in a total of 6400 acquisitions over an experimental time of nearly 75 minutes (see Table S3†); on the other hand, the ultrafast version with 1024 scans was over six times faster (less than 12 minutes). Although dividing the total signal *via* spatial encoding lowers the signal-to-noise ratio (SNR) per scan, multiple ultrafast experiments can be repeated in the time equivalent to a single scan of the traditional experiment (factoring in multiple indirect points). Consequently, the sensitivity per unit time in ultrafast experiments is usually at least as good and may even surpass the same sensitivity of traditional experiments.^41^,^49^ In our measurements, the estimated sensitivity per unit time provided by the ultrafast approach relative to the traditional approach was between 1 and 2 (see Table S3†).

### Nuclear hyperpolarization

**Fig. 4:**
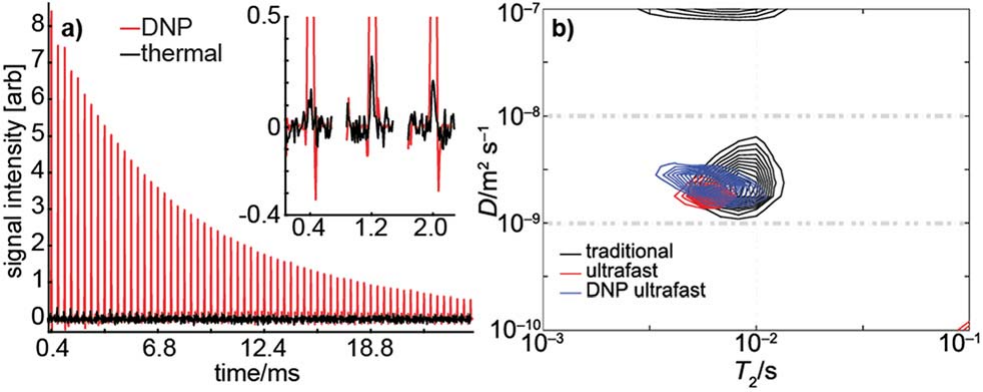
(a) Signal from a single DNP-hyperpolarized (red) scan and from 16 thermally polarized (black) scans in an ultrafast *D*–*T*_2_ measurement of water. Despite the small mole fraction of hyperpolarized water (0.003) and reduced number of scans, the hyperpolarized experiment results in a signal-to-noise ratio over 100 times greater than the thermal experiment. (b) A *D*–*T*_2_ map of hyperpolarized water measured in a single scan (blue). The map is in a good agreement with the corresponding traditional (black) and thermally polarized (red) ultrafast maps.

Using dynamic nuclear hyperpolarization (DNP), we were able to improve the NMR sensitivity of water by a factor of ∼10^5^ relative to thermal polarization in a 0.3 T magnet (see Fig. 4a). This sensitivity boost enabled us to measure a full *D*–*T*_2_ map of hyperpolarized water in a single acquisition using a single-sided magnet, regardless of the relatively low mole fraction (0.003) and concentration (170 mM) of hyperpolarized water (see Fig. 4b). Excluding time for preparation (∼20 min), delivery of the hyperpolarized water into an injection loop (∼1 s), and driving the sample into the sample holder (∼600 ms), the NMR experiment itself lasted only 22 ms. The resulting *D*–*T*_2_ map is in excellent agreement with those maps produced by traditional and ultrafast LNMR measurements of thermally polarized (pure) water. We emphasize that using hyperpolarization in the traditional experiment would, in practice, be impossible because it would require regeneration and delivery of DNP before the measurement of each indirect data point. Furthermore, any variability in the amount of hyperpolarization delivered before each indirect point would impose significant noise on the data.

The DNP data shown in Fig. 4 exhibits a SNR of ∼140. From conventional experiments, we estimate that a SNR of ∼20 is required for reliable extraction of *D* and *T*_2_ parameters. Therefore, the DNP experiment would be suitable for measuring diffusion and *T*_2_ in samples exhibiting an accessible volume fraction as low as ∼15%, even without increasing the concentration of hyperpolarized spins. Several types of materials, including zeolites, microporous polymers, and metal–organic frameworks may fall within this range. In addition, it is important to consider that DNP requires injection of the hyperpolarized liquid into the NMR volume. In our experiments, flow was stopped after injection using high pressure pumps actuated by pinch valves located at the inlet and outlet; these valves facilitate stabilization of the hyperpolarized fluid in the sample holder.^62^,^63^ Materials with low accessible volume-fractions and small pore sizes would benefit even further from injected DNP as they would require shorter stabilization times due to enhanced surface interactions.

Moreover, as shown in Fig. 4, DNP hyperpolarization allows the generation of strong NMR signals from hyperpolarized spins present even at low concentration. Therefore, the diffusion and adsorption behavior of dilute solutes in porous materials can potentially be studied with this method. The larger SNR afforded by hyperpolarization may also facilitate better discrimination of multiple, similar *D* values within a single sample as described elsewhere.^41^

## Conclusions

We demonstrated that spatially encoding diffusion to acquire simultaneous *D* and *T*_2_ data is feasible with a low-field, single-sided NMR instrument with a strong magnetic field gradient. The ultrafast *D*–*T*_2_ maps are in good agreement with traditional measurements, proving the reliability of the method. The results show that this method is applicable for a broad range of diffusion coefficients, at least from 10^–8^ to 10^–12^ m^2^ s^–1^. This method can also resolve multiple *D*–*T*_2_ peaks within a heterogeneous porous material, showing potential for more advanced chemical analysis. Significantly, the single-scan approach enables the use of hyperpolarization to dramatically boost experimental sensitivity. Using DNP hyperpolarization, we observed a sensitivity improvement of 10^5^ and measured a *D*–*T*_2_ map in a single-scan; this map shows excellent agreement with non-hyperpolarized measurements and was acquired despite a relatively low concentration of hyperpolarized water. This suggests ground-breaking prospects for low-field, advanced chemical analysis. To the best of our knowledge, this was the first application of nuclear spin hyperpolarization of protons with the single-sided NMR instruments (hyperpolarized ^129^Xe has been detected previously^30^). Although DNP is an expensive method requiring bulky and non-transportable facilities, other techniques such as PHIP and SEOP allow miniaturized,^60^,^64^ semi-portable hyperpolarization equipment, making mobile, hyperpolarized ultrafast LNMR an extremely attractive field.

## Conflicts of interest

There are no conflicts to declare.

## Supplementary Material

Supplementary information
