# Supplementary material for: Probing molecular dynamics with hyperpolarized ultrafast Laplace NMR using a low-field, single-sided magnet
Source: Chem Sci. 2018 Jun 28;9(28):6143–9. doi: 10.1039/c8sc01329b (PMC6053973; doi:10.1039/c8sc01329b)
Supplement: Supplementary file 1 [file SC-009-C8SC01329B-s001.pdf]

Electronic Supplementary Material (ESI) for Chemical Science.
This journal is © The Royal Society of Chemistry 2018

Electronic Supporting Information for

Probing molecular dynamics with hyperpolarized ultrafast Laplace NMR using a low-field, single-sided magnet

Jared N. King, Alfredo Fallorina, Justin Yu, Guannan Zhang, Ville-Veikko Telkki, Christian Hilty, and Tyler Meldrum

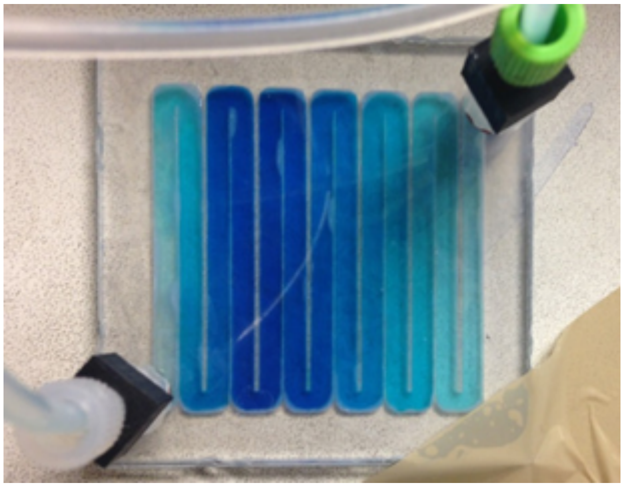

Figure S1. A sample holder made from Lexan used for DNP-hyperpolarized D–T_2 measurements of water. The s-patterned channels are 1.5 mm deep and span a 5 cm × 5 cm area, matching the area of the rf coil (total volume ~3 mL). The holder was placed within the sensitive region by supporting it on 4 mm glass slides on the magnet. The blue dye in the channel was not part of the D–T_2 experiment but was used to confirm an appropriate injection time for delivering the sample in the holder.

Table S1: Ultrafast experimental parameters

	ethylene glycol	water doped with copper (II) sulfate	glycerol	water in molecular sieves	DNP Water
**CHIRP pulse length [μs]**	477	127	2477	196.8	193.8
**CHIRP pulse power [kHz]**	48.1	48.1	32.1	24.1	16.7
**CHIRP pulse spatial bandwidth [μm]**	300	300	300	300	200
**dwell time per point [μs]**	4	4	4	2	6
**acquired points per echo**	152	78	78	72	54
**acquisition time per echo [μs]**	612	312	312	144	324
**number of echoes**	512	128	128	128	64
**echo time [μs]**	700	400	700	250	400
**repetition time [ms]**	1500	700	700	1000	400
**number of scans**	4096	1024	4096	8192	1
**Δ [ms]**	20	5	30	6	5
**δ [ms]**	1	0.3	5	0.4	0.4

Table S2: Traditional experimental parameters

	ethylene glycol	water doped with copper (II) sulfate	glycerol	water in molecular sieves
**acquired points per echo**	152	78	78	72
**dwell time per point [μs]**	4	4	4	2
**number of scans**	512	256	1024	1024
**number of 2D points**	21	25	21	25
**echo time [μs]**	700	400	700	250
**number of echoes**	512	128	128	128
**repetition time [ms]**	1500	700	700	1000
**Δ [ms]**	20	5	30	6
**δ [ms]**	1	0.3	5	0.4
**δ_{eff} [μs]**	0.1–988	0.1–287.8	0.1–4987.8	0.1–388

Table S3: T_2, D, and SNR comparison

Sample	T_1/ms	Meas. type	T_2/ms[a]	D/10^{-9} m² s⁻¹ [a]	SNR	N[b]	SNR/($N^{1/2}$)	UF gain[c]
**ethylene glycol**	285	TRAD	55 ± 16	0.2 ± 0.1	58.15	512	0.5608	
		UF	36 ± 6	0.17 ± 0.08	40.54	4096	0.6334	1.130
**water doped with copper (II) sulfate**	19	TRAD	8 ± 3	2 ± 1	70.20	256	0.8775	
		UF	6 ± 2	1.8 ± 0.4	57.38	1024	1.794	2.043
**glycerol**	31	TRAD	12 ± 8	0.0026 ± 0.0005	44.37	1024	0.3026	
		UF	8 ± 1	0.005 ± 0.002	23.47	4096	0.3667	1.212
**water in molecular sieves**	2500[d]	TRAD	20 ± 10<br>1.8 ± 0.3	2.6 ± 0.3<br>1.0 ± 0.5	19.95	1024	0.1247	
		UF	12 ± 4<br>1.1 ± 0.3	1.7 ± 0.3<br>2.0 ± 0.5	20.65	8192	0.2282	1.830
**DNP-hyperpolarized water**	2500[e]	UF	7 ± 2	2.2 ± 0.8	139.1	1	139.1	—

[a]The errors in T_2 and D are maximum errors, determined from the width of the peaks on a logarithmic scale, which is the scale used for ILT.
[b]Number of acquired scans, not factoring in multiple indirect points for the traditional acquisitions
[c]Calculated using an SNR/($N^{1/2}$) basis
[d]T_1 of bulk water, not necessarily that of water trapped in the sieves
[e]T_1 of DI water, irrelevant for hyperpolarized samples
